# Supplementary material for: Potent Antitrypanosomal Activities of 3-Aminosteroids against African Trypanosomes: Investigation of Cellular Effects and of Cross-Resistance with Existing Drugs
Source: Molecules. 2019 Jan 12;24(2):268. doi: 10.3390/molecules24020268 (PMC6359104; doi:10.3390/molecules24020268)
Supplement: Supplementary file 1 [file molecules-24-00268-s001.pdf]

Supplementary material

Potent Antitrypanosomal Activities of 3-Aminosteroids against African Trypanosomes: Investigation of Cellular Effects and of Cross-Resistance with Existing Drugs

Charles O. Nnadi [1,2], Godwin U. Ebiloma [3,4], Jennifer A. Black [5,6], Ngozi J. Nwodo [2], Leandro Lemgruber [3], Thomas J. Schmidt [1,*] and Harry P. de Koning [3]

1 Institute of Pharmaceutical Biology and Phytochemistry (IPBP), University of Münster, Pharma Campus Corrensstraße 48, D-48149 Münster, Germany; charles.nnadi@unn.edu.ng
2 Department of Pharmaceutical and Medicinal Chemistry, Faculty of Pharmaceutical Sciences, University of Nigeria Nsukka, 410001 Enugu, Nigeria; ngozi.nwodo@unn.edu.ng
3 Institute of Infection, Immunity and Inflammation, College of Medical, Veterinary and Life Sciences, University of Glasgow, Glasgow G12 8TA, UK; godwin4godwin@gmail.com (G.U.E.); Leandro.LemgruberSoares@glasgow.ac.uk (L.L.); Harry.De-Koning@glasgow.ac.uk (H.P.d.K.)
4 Department of Applied Biology, Kyoto Institute of Technology, Kyoto 606-8585, Japan
5 The Wellcome Trust Centre for Molecular Parasitology, Institute of Infection, Immunity and Inflammation, University of Glasgow, Glasgow G12 8TA, UK; jennifer.stortz@glasgow.ac.uk
6 Department of Cell and Molecular Biology, Ribeirão Preto Medical School, University of São Paulo, Ribeirão Preto 14049-900, Brazil
* Correspondence: thomschm@uni-muenster.de; Tel.: +49-251-83-33378

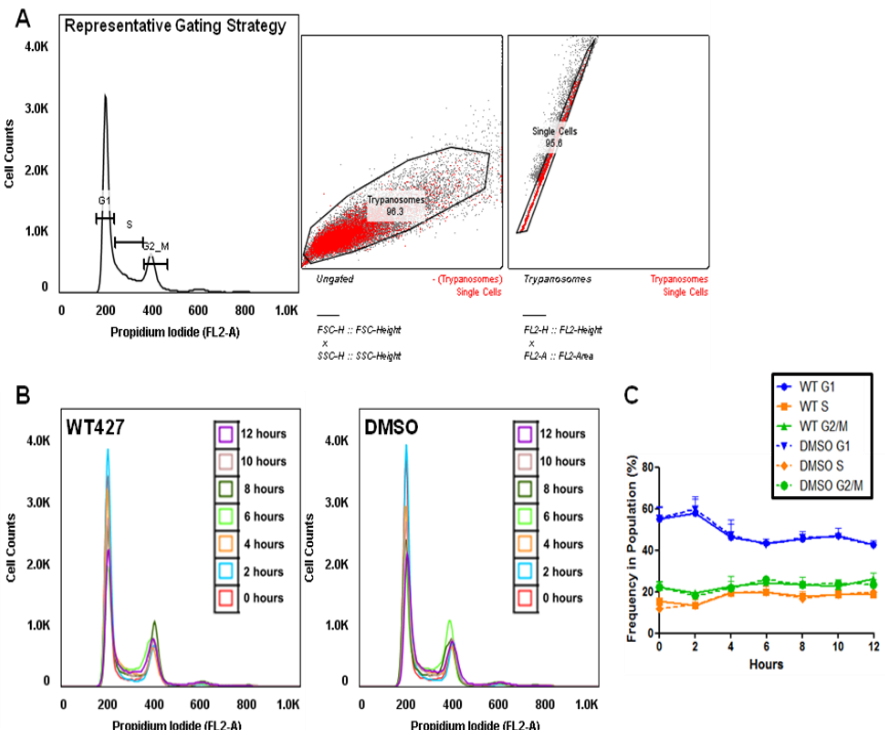

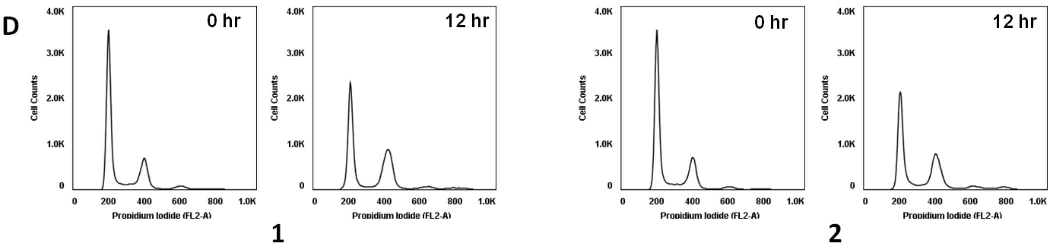

Figure 1. (**A**) Representative gating strategy to analyse the DNA content of BSF *T. brucei* cells. FSC-H and SSC-H were used to identify cells required for analysis and the FSC-A plotted against the FSC-H was used to exclude doublet cells. The FL2-A channel was used to detect propidium iodide signal and the subsequently gated data plotted as histograms. The cell cycle stages corresponding to each peak or the histogram are marked on the first plot and their positions applied to all subsequent samples. (**B**) Representative cell cycle profiles of WT Lister 427 BSF cells and WT Lister 427 cells treated with DMSO (0.09% v/v). Data shown from one experiment as overlaid histograms. Three biological replicates were performed. Over 40000 events were analysed per experiment for each condition. (**C**) Graphs depicts DNA content collected by flow cytometry. The percentage of each cell cycle stage were plotted as a frequency of the total cell population (%) over 12 h. Legend is as shown above the graph; solid line = WT Lister 427 cells, dashed line = DMSO treated WT Lister 427 cells, error bars = ± SD (n = 3). (**D**) Representative cell cycle profiles of WT Lister 427 cells treated with compounds 1 and 2 (concentration >IC_{50}) from one experiment as histogram.
